# Research on the road: Partnering with community emergency medical services to expand access to clinical trials

**DOI:** 10.1017/cts.2026.10761

**Published:** 2026-05-29

**Authors:** Laurel O’Connor, Alex Ulintz, Joel Rowe, Peter K. Lindenauer, Apurv Soni

**Affiliations:** 1 Emergency Medicine, University of Massachusetts Chan Medical School, Worcester, MA, USA; 2 Emergency Medicine, The Ohio State University Medical Center, Columbus, OH, USA; 3 Emergency Medicine, University of Florida Academic Health Center, Gainesville, FL, USA; 4 Healthcare Delivery and Population Sciences and Department of Medicine, University of Massachusetts Chan Medical School Baystate, Springfield, MA, USA; 5 Department of Medicine, UMass Chan Medical School, Worcester, MA, USA

**Keywords:** Pragmatic clinical Trials, decentralized clinical trials, community paramedic, mobile integrated health, mobile health

## Abstract

Traditional site-based clinical trials impose substantial logistical burdens that limit participation among individuals with geographic, mobility, transportation, and socioeconomic constraints, thereby undermining research equity and generalizability. Although pragmatic and decentralized trial designs seek to address these barriers, many health systems lack a mobile clinical workforce capable of conducting in-person research activities in community settings. Mobile Integrated Health (MIH) and Community Paramedicine (CP) programs, which leverage community paramedics and EMTs (MIH-CPs) to provide field-based care, may provide scalable infrastructure to support decentralized research delivery. We describe an institutional model that cross-trains and integrates MIH-CPs into research teams. Using study protocols, training materials, operational procedures, and implementation documents from five studies, we analyzed staffing models, credentialing and training processes, workflow integration, fidelity monitoring, and ethical and safety considerations. The included studies spanned pragmatic randomized and hybrid trials, and observational cohort studies across diverse clinical populations. MIH-CPs conducted recruitment, consent, assessments, intervention delivery, and data collection. Structured training, defined communication pathways, and integration with established clinical workflows supported protocol fidelity, participant safety, and operational feasibility. Embedding mobile community-based clinicians within research teams represents a pragmatic, equity-oriented strategy to expand decentralized trial capacity, reduce participant burden, and accelerate translation of evidence into real-world practice.

## Introduction

Clinical trials are a cornerstone of biomedical investigation and remain the gold standard for validating procedures, therapeutics, and health system interventions [1,2]. Despite this foundational role, traditional site-based clinical trial models impose substantial burdens on participants and often fail to reflect the populations most affected by disease [3,4]. Requirements for frequent in-person visits, rigid scheduling, and travel to centralized research sites disproportionately exclude individuals with mobility limitations, transportation barriers, socioeconomic constraints, chronic illness burden, limited health literacy, immunocompromised status, or geographic isolation [3–5]. In a study of randomized trials approved by ethics committees, 31.1% were discontinued, with poor recruitment accounting for 45.4% of discontinuations [6]. Despite substantial investment in clinical research infrastructures, only 2–5% of eligible patients actually participate in relevant clinical trials, and up to 80% of trials are delayed by poor recruitment [3,7–9]. At the population level, patients in rural regions have 77% lower odds of invitation than urban residents [10]. These structural barriers have contributed to persistent inequities in trial participation, constraining the efficiency and generalizability of clinical research findings [5].

Decentralized and hybrid trial models have been proposed to overcome selection bias related to access and awareness of clinical trial sites, and to reduce participation burden by shifting selected trial activities away from academic sites and toward participants and local care settings [11–13]. Such approaches introduce operational demands related to staffing, oversight, terminology, and data quality that require explicit design and governance [14,15]. To support staff coverage for decentralization, teams have increasingly incorporated non-traditional study personnel, including “mobile” assets, such as community health workers and peer navigators, with the goal of extending trial reach and strengthening trust between investigators and communities [16–19].

Mobile Integrated Health and Community Paramedicine (MIH-CP) programs that leverage community emergency medical services personnel, including community paramedics (CPs) and community emergency medical technicians (C-EMTs) [20], together referred to as MIH-CPs, may represent a powerful and underutilized solution for decentralizing clinical trials that require in-person clinical interactions. MIH-CPs are embedded in communities, inherently mobile, and are capable of bringing study procedures directly to participants in homes, workplaces, shelters, schools, and congregate living environments [20]. They already have a well-established clinical role and are progressively being integrated into health systems to improve access to care and clinical outcomes in high-risk patients [20]. In some institutions, including our own, they are also being integrated into clinical research studies [21,22]. The goal of this project is to describe core components and special considerations for studies that leverage this workforce to expand access to research participation. By leveraging their mobility, clinical skills, and community embeddedness, MIH-CPs have the potential to enhance trial accessibility, equity, adherence, and safety while simultaneously strengthening the translational continuum from evidence generation to real-world implementation.

## Methods

### Research setting and context

This manuscript describes an institutional approach to incorporating MIH-CPs as clinical research personnel within decentralized and hybrid clinical studies that require in-person assessments, monitoring, or protocolized intervention delivery. UMass Memorial Health is based on an urban academic tertiary care medical center that serves urban, suburban, and rural communities. The system handles over 1.3 million annual ambulatory visits and approximately 260,000 emergency department encounters. The medical center has an affiliate MIH program overseen by the Division of Emergency Medical Services that performs preventative, acute, and transitional MIH-CP services as part of the clinical standard of care. The MIH program is fully licensed under the state Department of Public Health and is overseen by board-certified EMS physicians. The program, which is part of a third-service hospital-based EMS agency, currently has six full-time paramedics, two part-time paramedics, one EMT, four medical directors, and two fellows, and performs approximately 750 home visits annually.

Relevant studies were identified through a structured internal review process conducted by the study team. The primary data source for study identification consisted of operational records maintained by the institutional MIH program, which track and document all deployments of MIH-CPs, including personnel involvement in research activities. These records are maintained to track program resources, ensure regulatory compliance, and preserve a clear delineation between the operational and financial aspects of research-related activities and routine clinical operations.

The initial list of candidate studies abstracted from operational records was subsequently validated through triangulation with MIH program leadership and institutional principal investigators to enhance completeness, reduce misclassification, and confirm study capture. Final eligibility determinations were made through structured review of study protocols and supporting documentation. Studies were eligible for inclusion if at least one CP-MIH clinician engaged in human subjects research activities as part of the study protocol, and the study was either ongoing or completed within the last two years. Observational studies were excluded when MIH–CP clinicians provided only standard-of-care clinical care without an investigational research role. The resulting exemplar studies encompassed multiple study designs, including pilot studies, pragmatic randomized trials, hybrid effectiveness–implementation studies, and observational cohort studies, and spanned a range of clinical domains. Study procedures were conducted in diverse community-based settings, including private residences, assisted and supported living facilities, and other non-clinical environments aligned with the needs of the target populations.

A multidisciplinary team of four clinician-scientists with expertise in clinical trial leadership systematically reviewed trial protocols, training materials, standard operating procedures, and implementation documentation from the included studies. Using this structured review, we identified and characterized core design elements, staffing and supervision models, training requirements, regulatory considerations, and clinical and operational workflows that enable community MIH–CP clinicians to function as research personnel. The primary objective is to describe the roles, competencies, supervision structures, and procedural safeguards required to support MIH–CP participation in research while maintaining protocol fidelity, licensure compliance, and participant safety. Secondary objectives include describing strategies for integrating research activities into existing clinical care workflows, outlining ethical and regulatory considerations unique to community-based research delivery, and articulating the rationale and perceived advantages of incorporating MIH–CP clinicians into study teams. This study was determined to be “Not Human Subjects Research” by the IRB of the affiliate medical school.

## Results

**Figure 1.**
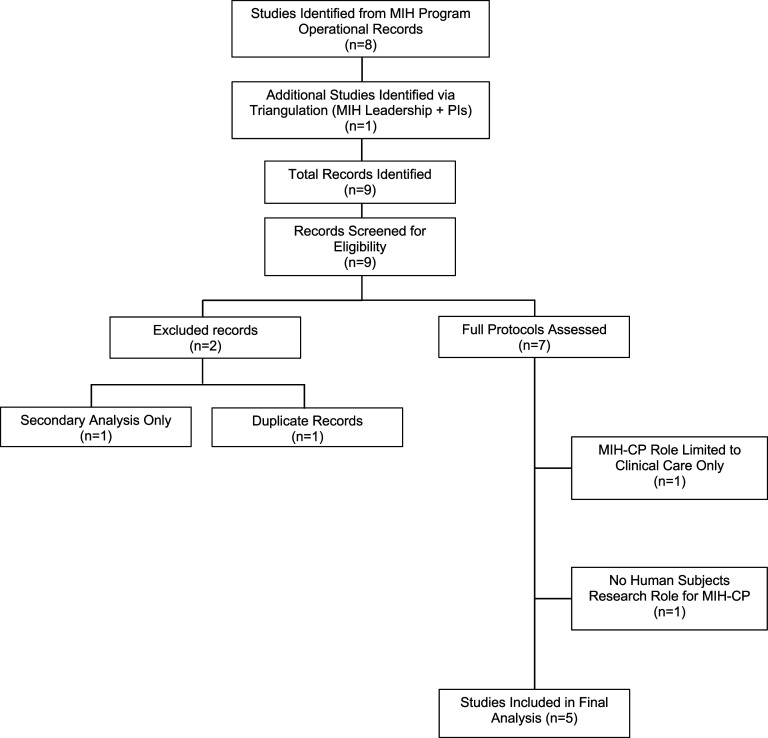
Internal study identification and eligibility flow diagram. This figure illustrates the structured internal process used to identify, screen, and determine eligibility of studies within the institution. Study identification was conducted using MIH operational records. Candidate studies were validated through triangulation with MIH program leadership and institutional principal investigators. Eligibility was determined through review of study protocols and supporting documentation based on predefined inclusion and exclusion criteria, including MIH-CP involvement in human subjects research activities, study timeframe, and relevance to eligible study designs.

Figure 1 illustrates the workflow used to identify, screen, and determine the eligibility of studies for inclusion in this project (*n* = 9). Table 1 describes the final list of included studies (*n* = 5), including their design and outcomes, the study team composition, and the role of MIHCPs. **Supplemental File 1** summarizes the rationale, approach, and available findings for each study.

**Table 1.** Example clinical research studies utilizing CP research staff Table 1 long description.

Study	Design	Setting & participants	MIH-CP research activities	MIH-CP Clinical role	Primary outcomes	Rationale for MIH-CP integration
Paramedic Evaluation for Acute COPD Exacerbation (PEACE Trial) (NCT07072039)	Pragmatic randomized controlled trial (hybrid decentralized)	Adults with COPD recently discharged from ED or hospital following exacerbation	Recruitment; informed consent; data collection	Post-discharge home visits; on-demand evaluation for acute COPD symptoms	ED utilization; hospitalization; 30-day rehospitalization; mortality; CAT score	Delivery of home-based clinical assessments and interventions; reduced participant burden; enabled rapid response to acute symptoms
Healthy at Home for COPD (NCT06000696)	Pilot nonrandomized clinical trial (decentralized)	Adults with COPD at high risk for exacerbation	Data collection	On-demand in-home evaluation for acute respiratory symptoms	ED utilization; hospitalization; mortality; 30-day rehospitalization; CAT score	Supported participants with limited digital literacy; enabled in-home clinical assessment integrated with remote monitoring
Paramedic Acute Response Approach for Adults with Intellectual and Developmental Disabilities (PARA-AIDD)	Observational cohort study	Adults with intellectual and developmental disabilities in supported residential settings	Recruitment; Data collection	On-demand evaluation for acute undifferentiated illness	ED utilization; hospitalization; claims-based cost outcomes; difference-in-differences analysis	Improved access to acute assessment in populations with communication and sensory barriers; support for caregivers and residential staff
Paramedic Assisted Community Evaluation After Discharge (PACED)	Observational cohort study	Frail older adults discharged from inpatient hospitalization	Data collection	Post-discharge transitional care home visits; flexible on-demand dispatch at discharge	30- and 90-day rehospitalization	Enabled in-home assessment of clinical stability, safety, and cognition during high-risk transitional period
Subacute Rehabilitation at Home (SARAH) (NCT06547645)	Pragmatic randomized controlled trial (hybrid decentralized)	Older adults discharged from inpatient hospitalization requiring skilled nursing care	Recruitment; informed consent; data collection	Support for home-based skilled nursing-level care team; in-home assessments; on-demand dispatch for acute needs	30-, 60-, and 90-day rehospitalization; patient satisfaction; cost-effectiveness	Supported decentralized trial operations across post-acute setting; enabled longitudinal follow-up and integration of clinical assessment with home-based care team

Across the five included studies, we identified two distinct patterns of MIH-CP research integration, differentiated by the primary role of the MIH-CP within the study. In the first model, MIH-CPs functioned primarily as clinical interventionists, with research activities embedded within care delivery. In studies such as the PEACE trial, Healthy at Home for COPD, and PARA-AIDD, MIH-CPs delivered protocolized, in-home clinical care aligned with traditional EMS scope of practice, including the evaluation and management of acute illness and exacerbations of chronic disease. In this model, research tasks, such as recruitment, informed consent, and data collection, were integrated into these clinical encounters, but the central function of the MIH-CP remained the delivery of clinical care.

In the second model, MIH-CPs functioned primarily as mobile research personnel, with clinical capabilities augmenting trial execution. In studies such as SARAH and PACED, MIH-CPs were deployed to support core research operations, including participant recruitment, longitudinal follow-up, and in-home data collection across post-acute and transitional care environments. These roles extended into settings where EMS personnel have not traditionally been involved and were structured around enabling decentralized trial workflows rather than delivering a discrete EMS-aligned clinical intervention. While MIH-CPs retained the ability to perform clinical assessments and respond to acute needs, their primary function in these studies was to serve as a flexible, community-based research workforce capable of bridging gaps in access, continuity, and data capture across fragmented care settings.

### EMS clinician eligibility, credentialing, and training

All participating community MIH-CP clinicians entered their research role with foundational EMT-B or paramedic-level education and certification. MIH-CPs received additional institution-specific training in community EMS protocols, including advanced patient assessment techniques, chronic disease management, medication reconciliation, performing non-clinical drivers of health screening, and documenting in the institutional electronic health record (EHR), which aligned with their clinical duties. MIH-CPs who cross-train as research staff also receive the standard institutional training required for staff engaged in human subjects research at the coordinator or research assistant level. This includes instruction in human subjects research protections and completion of required online modules through the Collaborative Institutional Training Initiative (CITI) program. MIH-CP clinicians are also required to shadow an experienced researcher coordinator for a minimum of 1 month. All MIH-CP clinicians are also trained on study-specific software platforms (e.g., REDCap or MyDataHelps) for data collection. Study-specific training is administered by the principal investigator (PI) for each protocol alongside non-clinical personnel functioning as research coordinators or research assistants. Table 2 summarizes the components of MIH-CP clinician training.

**Table 2.** Example clinical research studies utilizing CP research staff

Training domain	Training type	Description	Oversight
Clinical	Foundational Clinical Certification	EMT-B or paramedic education and active certification, regulated at the state level through the Department of Public Health and national level through the national registry of emergency medical technicians	MIH Medical Director State EMS authorities
	Institution-Specific Community EMS Training	Advanced patient assessment, chronic disease management, medication reconciliation, and screening for non-clinical drivers of health.. All personnel also train on the institutional EHR with the user-role of “MIH Paramedic.”	MIH program leadership
Research	Human Subjects and GCP Training	Training included human subjects research protections, Good Clinical Practice (GCP), and required online modules through the Collaborative Institutional Training Initiative (CITI) program.	Institutional research compliance office
	Research Staff Core Training	Standard institutional training required for clinical trial staff at the coordinator or research assistant level. One month of shadowing an experienced research coordinator in the affiliate lab	Principal investigator (PI)
	Research Data Systems Training	Training on study-specific electronic data capture platforms (e.g., REDCap, MyDataHelps)	Research coordination team
	Protocol-Specific Training	For each study, protocol-specific training is delivered by the PI alongside non-clinical research coordinators or research assistants.	Principal investigator (PI)

### Study team structure and communication

MIH-CPs are employed by the EMS agency and are members of the agency’s collective bargaining unit, and dual onboarded as research coordinators through the affiliate medical school. Their research effort is supported through medical school funding from individual study PIs. Employment models varied: some MIH-CPs were funded at 100% effort as part of the research team, whereas others had hybrid roles, combining research responsibilities with dedicated clinical shifts. During hybrid shifts, paramedics were permitted to contribute to the alternate domain if operationally feasible; however, work priority was determined by the designation of the shift, with clinical responsibilities taking precedence during clinical shifts and research activities prioritized during research-designated time.

Across studies, MIH-CPs were integrated into multidisciplinary research teams that included a principal investigator (PI), co-investigators (Co-Is), a physician medical director overseeing MIH operations (who typically also serves as a CO-I or PI), research coordinators, and relevant clinical partners (e.g., primary care or specialty clinicians). MIH-CPs primarily conducted in-home research activities, including clinical assessments, protocolized interventions, and data capture, while non-clinical research coordinators led participant outreach, recruitment, consent scheduling, and centralized data management. In select studies, MIH-CPs performed recruitment and consent procedures in participants’ homes.

**Figure 2.**
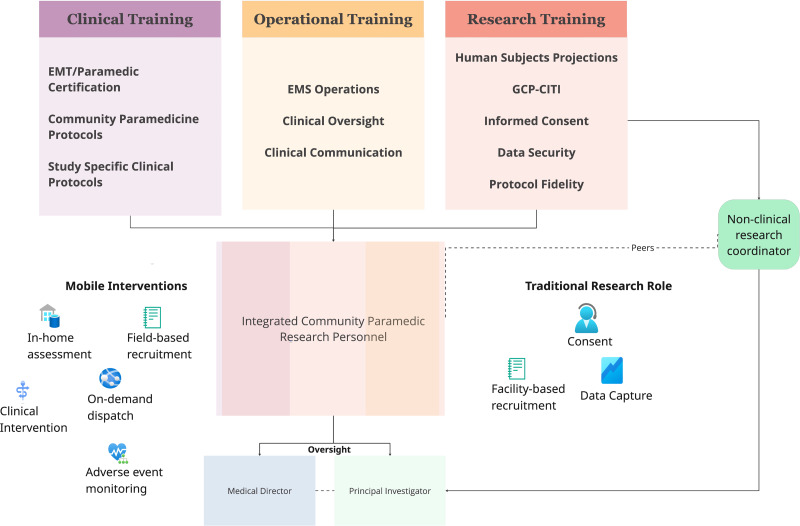
Figure 2 long description. Research team structure with MIH-CP clinicians: MIH-CP clinicians have a wide breadth of training that enables them to perform both traditional research activities and clinical interventions. They are often integrated into research teams alongside non-provider research coordinators and receive joint oversight from both a medical director, for clinical concerns, and a principal investigator, for research matters.

Research oversight is provided by the PI, who is responsible for protocol training, monitoring adherence to study procedures, and ensuring regulatory compliance. Trial-specific communication pathways are always established for MIH-CPs and documented in the study protocol; these include mechanisms for real-time consultation with patients’ usual clinical teams before, during, or after study visits as needed (e.g., telephone or secure telehealth platforms) and the designated medical director. Delivery of clinical interventions, diagnostic results, and medication administration was documented in both the institutional EHR and study-specific data capture systems. Regular interdisciplinary meetings, including MIH-CP staff, non-clinical research staff, investigators, and the medical director, are held periodically at predetermined intervals to review operational issues, protocol deviations, safety events, and workflow challenges. Figure 2 depicts the roles and team structure of study teams that include community EMS personnel.

### Date management and integration with clinical care workflows

Research activities conducted by MIH-CP clinicians are intentionally aligned, when feasible, with existing clinical care workflows to minimize operational disruption, reduce cognitive burden for both clinical and research staff, and preserve continuity of care. Study protocols are designed to integrate with routine operational processes, including established referral pathways (e.g., EHR-based orders), documentation standards, and clinical escalation procedures. When feasible, study-triggered visits are structured to mirror standard MIH clinical encounters in scope, content, and documentation. Study encounters are documented in the institutional EHR using note templates consistent with clinical practice, with explicit labeling to denote research-related activity. Strategies used to support clinical workflow integration are summarized in Table 3.

**Table 3.** Special considerations in studies leveraging community EMS personnel

Domain	Component	Description	Example
**Clinical workflow integration**	Alignment with routine care	Research activities conducted by MIH-CP staff are aligned with existing clinical care workflows to minimize disruption and support continuity.	PARA-AIDD: the PARA-AIDD template is designed to replicate the documentation format used by group homes for “sick visits,” so the encounter form serves as their mandated documentation of the visit.
	Referral pathways	Study protocols leverage routine operational processes, including EHR-based referral and order pathways used for standard MIH clinical encounters.	PEACE: primary care and subspecialist clinicians can request acute/unplanned visits for enrolled participants via the existing EHR order for MIH services
	Visit structure	When feasible, study-triggered visits mirrored standard MIH clinical encounters in structure, content, and clinical scope	Healthy at Home: unplanned visits are documented using a note-template co-designed by the pulmonary and MIH teams for standard of care visits outside of research contexts.
	Documentation standards	Study encounters are documented in the EHR using note templates consistent with routine clinical practice, with clear labeling to identify encounters as research-related.	PEACE: participants’ pulmonologists receive electronic copies of the templated PEACE research (non-acute) note. Red font at the top of the note delineates it as a research visit.
**Care team communication**	Clinician notification	For studies involving research-specific interventions, patients’ primary care and specialty clinicians were notified at the time of enrollment; most were familiar with MIH operations within the health system.	PCPs are notified at the time of their patient’s hospital discharge via direct EHR secure chat from the research MIH-CP clinicians that their patients would be receiving a home visit as part of the PACED program.
**Safety and escalation**	Emergency escalation	If a participant experiences a medical emergency during a study visit, care is escalated to the standard 9-1-1 EMS system, with a separate, non-study EMS unit dispatched to transport the patient.	PEACE clinical trial: unplanned/acute research visits are escalated to traditional 911 services if patients had serious or complex medical needs at the time of the research visit that cannot be safely managed by the MIH-CP clinician
**Data collection and management**	Data types	MIH-CPs collect quantitative and qualitative data during in-home visits, including demographics, clinical assessments functional measures, and medication reconciliation	PEACE: MIH-CPs performed a proctored six-minute walking test and 1 minute sit to stand assessment in the home during study visits.
	Data collection tools	Data is entered into HIPAA-compliant systems (e.g., REDCap, MyDataHelps) employing role-based access controls.	Healthy at Home: MIH-CPs could view the MyDataHelps study platform to review clinical data patient during research visits.
**Fidelity monitoring**	Standardized audit tools	Fidelity was monitored using structured case report forms, visit checklists, and required documentation fields. Protocol deviations, were recorded as implementation outcomes.	PEACE has a standard home-visit audit checklist performed by the PI after each home visit; protocol fidelity is a primary trial-outcome.
	Real-time prompts	Embedded documentation tools prompted completion of core study procedures and facilitated real-time identification of omissions or deviations.	The PARA-AIDD visit form contains smart-data elements for the documentation of required study procedures that are automatically populated into a study report. The note cannot be signed until every element is completed
**Ethics and human subjects protections**	Preventing therapeutic misconception/ role confusion	Research activities explicitly distinguished from routine clinical care in protocols, consent forms, and clinician- and participant-facing materials	Healthy at Home: Educational materials clearly distinguish “Your Medical Care” from “Optional Research Activities,” emphasizing that research participation is voluntary and separate from clinical care.
	Equitable access to care	Patients are offered MIH–CP standard-of-care services regardless of study enrollment	PEACE: Patients who declined to participate in research activities were still offered MIH visits through standard pulmonary clinic protocols
	Informed decision-making	Participants informed of potential costs, including whether MIH–CP services were billable or study-covered	Healthy at Home: The consent form specifies potential out-of-pocket costs and distinguishes billable clinical services from study-funded research activities.
	Study staff safety	Study protocols address protocols for staff experiencing personal safety concerns during home visits	PEACE Study: per protocol MIH-CPS communicate verbally with their dispatcher any time they enter a patient’s home

For studies involving investigational interventions, participants’ primary care and relevant specialty clinicians were notified at the time of enrollment; in most cases, these clinicians were already familiar with MIH operations within the health system. During study visits, if a participant is identified as experiencing a medical emergency, care is immediately escalated through the standard 9-1-1 EMS system, and a separate, non-study EMS unit is dispatched for transport to the nearest appropriate emergency department in accordance with established emergency care protocols; this process mirrors care escalation during standard of care MIH visits.

### Data collection and management

Data collection procedures are tailored to the requirements of each trial. MIH-CPs collected both quantitative and qualitative data during home visits, including demographic information, clinical assessments, vital signs, functional measures, laboratory results, patient-reported outcomes, and documentation of protocolized interventions. Data collection tools included structured case report forms, validated survey instruments, and electronic data capture platforms. All research data are entered into secure, Health Insurance Portability and Accountability Act (HIPAA)–compliant systems (e.g., REDCap, MyDataHelps) with role-based access controls. Procedures for data quality monitoring, audit trails, and version control followed standard institutional research practices. Data management strategies and examples are summarized in Table 3.

### Fidelity monitoring procedures

Protocol fidelity is monitored using a combination of training verification, standardized documentation, and ongoing oversight throughout study implementation and execution. Prior to participating in research activities, MIH-CPs complete study-specific competency assessments to ensure understanding of protocol requirements, data collection procedures, and escalation pathways. During trial conduct, fidelity to study protocols is assessed through structured case report forms, visit checklists, and required documentation fields embedded within electronic data capture systems. These tools prompted completion of core study procedures and facilitated real-time identification of omissions or deviations. Research coordinators and PIs conduct routine reviews of submitted documentation to verify adherence to protocol-defined activities. A description of protocol fidelity strategies and examples is summarized in Table 3. Deviations from study protocols, including missed procedures, delayed visits, or unplanned clinical escalations, are recorded and reviewed by the study team as implementation outcomes. Regular interdisciplinary study staff meetings and data safety monitoring board meetings provided a forum to review fidelity metrics, discuss operational challenges, and implement refinements as needed.

### Ethical considerations and safety monitoring

Table 3 summarizes special ethical considerations relevant to the use of MIH-CP clinicians in clinical research protocols. Because MIH-CPs routinely provide direct non-research patient care in the home, all study procedures were explicitly defined, communicated, and documented as research interventions, distinguishing them from extensions of routine clinical services in all clinician- and participant-facing materials. Finally, MIH-CPs also commonly work in environments where safety risks may be unpredictable, including unstable housing, geographically isolated settings, or with patients experiencing behavioral health crises. All research protocols included training that addressed field safety, access to supervision, and pathways for rapid consultation with the PI or medical director. Risk mitigation strategies, including co-responding personnel when appropriate, pre-visit safety screening, and secure communication tools, are considered required institutional components of ethical decentralized trial design involving mobile assets.

### Integration challenges

Integrating MIH-CP clinicians as research staff introduced some operational and conceptual challenges, most notably related to role delineation, communication, and funding. In some cases, ambulatory clinicians and MIH–CP staff were uncertain about overlapping responsibilities when protocolized research visits closely resembled standard ambulatory clinical encounters. This occasionally led to perceptions of duplicative effort or uncertainty about who was responsible for clinical follow-up. For example, when MIH–CP clinicians identified clinically actionable findings during research visits- such as medication adherence concerns-it was not always immediately clear whether responsibility for treatment changes or follow-up rested with the research team, the clinical MIH team, or the patient’s ambulatory provider, underscoring the need for explicit role definitions and communication pathways.

In parallel, delineating clinical communication from research communication proved challenging in home-based settings, where protocolized research activities frequently generated clinically relevant findings. Clear guidance is required to determine when information should remain within research documentation versus when escalation through clinical channels is appropriate, to avoid both under-communication and inappropriate clinical action based on research-only data. Some of this confusion was related to cultural change for the MIH-CPs from a role that is clinical to one with dual purposes.

An additional challenge involved funding MIH-CP clinicians in dual clinical and research roles. Traditional funding mechanisms separate reimbursable clinical care from grant-supported research activity, creating complexity when MIH-CP clinicians simultaneously deliver protocolized research interventions and provide clinical assessment or treatment. This required careful effort allocation, transparent role definitions, and coordination across research administration, clinical operations, and finance teams to ensure compliance with regulatory and billing requirements. Other challenges included the cognitive burden of navigating dual roles, maintaining protocol fidelity in dynamic community environments, and coordinating schedules across mobile clinicians, participants, and centralized research teams. Addressing these challenges necessitated iterative workflow refinement, standardized documentation practices, and ongoing interdisciplinary communication.

## Discussion

In this project, we described the institutional use of cross-trained MIH-CP clinicians as key personnel on clinical research teams for decentralized and hybrid trial designs requiring in-person clinical assessments, evaluation, and treatment. Across the sample of trials included in the analysis, spanning COPD care, transitional support for older adults, and acute care for adults with disabilities, we describe the feasibility of MIH-CPs as research staff and describe a potential approach to reproducing this unique team structure. This work builds upon early case reports of clinical trials that leverage EMS personnel as research staff [23,24].

Regulators anticipate that decentralized trial elements can reduce participation burden and improve real-world representativeness, but they also emphasize concerns about investigator oversight and participant safety when physical examinations and face-to-face contact are limited [15]. This tension is particularly relevant for interventions that require in-person assessment or monitoring, which has motivated hybrid designs and the use of mobile clinicians [11,15]. The MIH-CP approach described here aligns with this hybrid direction by pairing remote data capture with home-based clinical assessment and escalation pathways. As a trusted, mobile clinical workforce embedded in homes, neighborhoods, and nontraditional care environments, they are well positioned to deliver interventions, monitor safety, ensure follow-up, and facilitate bidirectional information flow between participants, research teams, and clinical providers. By bridging standard-of-care practice with trial operations, MIH–CPs may also establish a foundation to smooth future implementation, leveraging existing personnel whose daily work is already designed for mobile, community-based care. This dual function aligns closely with CTSA priorities, emphasizing equity, community partnership, and real-world translation.

We noted two distinct but complementary models for integrating MIH-CPs clinicians into clinical research: a care delivery-anchored model, in which research activities are embedded within traditional EMS-aligned clinical interventions, and a research infrastructure–anchored model, in which MIH-CPs function as a mobile workforce supporting decentralized trial operations outside of traditional EMS duties. The former leverages MIH clinical capabilities to extend protocolized care into the home while incorporating research procedures, representing a natural evolution of established CP roles. In contrast, the latter reflects a more substantial expansion of MIH-CP function, positioning these clinicians as flexible, community-based research personnel capable of supporting recruitment, longitudinal follow-up, and data collection across a broader range of care environments. This second model may be particularly well suited to decentralized and hybrid trial designs, where fragmentation of care settings and limited access to in-person research infrastructure remain persistent barriers.

Expanding the role of MIH-CPs into clinical research roles also broadens the depth and scope, and diversity of professional opportunities for prehospital clinicians. Prehospital providers face well-documented challenges related to burnout, limited career advancement pathways, and high turnover, often driven by the physically demanding and episodic nature of traditional emergency response work [25,26]. Integrating EMS personnel into clinical trial teams introduces a new, intellectually engaging professional domain that leverages their clinical expertise, community familiarity, and independent decision-making skills. Participation in research activities broadens the scope of paramedic practice while fostering professional identity, job satisfaction, and long-term career sustainability; previous literature has shown evidence that prehospital clinicians are increasingly engaged with and interested in clinical research as a way to improve patient care delivery [27]. Previously identified barriers to incorporating prehospital clinicians into research roles have primarily involved insufficient time and limited training [27]. Creating structured pathways for EMS clinicians to contribute to research not only enhances trial capacity but also supports workforce stability by offering varied, rewarding roles that recognize and build upon existing prehospital clinician competencies.

Importantly, this case study is limited to a single institution and describes a single operational approach. The model described in this project is embedded within a health system; however, MIH-CP programs nationally are highly heterogeneous in their operational and organizational structures, spanning fire–based systems, private EMS agencies, municipal services, and health system–embedded models with no single model representing a clear operational majority [28]. This contextual difference may influence implementation pathways, access to clinical and research infrastructure, and integration with existing health system workflows, and should be considered when interpreting the transferability of these findings. Prior literature describes MIH-CP as a highly context-dependent model that is shaped by local workforce organization, funding mechanisms, and health system integration pathways [28]. These structural differences may meaningfully influence program capabilities, alignment with research infrastructure, and the feasibility of adequate integration with downstream healthcare services. As such, the findings of this study may not be fully generalizable to operationally distinct MIH-CP models without adaptation to local organizational and regulatory contexts.

Implementing any MIH-CP model requires thoughtful alignment of training, workflows, and ethics. Cross-training MIH-CP personnel in research coordination, human subjects protection, and study-specific procedures ensures protocol fidelity, while clear delineation between research and clinical care helps avoid role confusion for participants and clinical teams. Robust communication pathways between MIH-CPs, investigators, and primary clinical teams are essential, particularly when paramedics detect clinical decline or safety concerns during home visits. Safety considerations, including field conditions, clinician well-being, participant privacy, and engagement with patients’ primary teams, must remain central when deploying research personnel into community environments. Future research should examine approaches to the use of MIH-CPs from agencies external to the health system (e.g., fire-based or private services) as research personnel. They should also evaluate the comparative impact of MIH-CP-supported decentralized trials versus other mobile personnel models, evaluate cost implications, and explore MIH-CP clinical roles in later-stage implementation studies.

These findings situate MIH within the broader landscape of U.S. translational science. Despite substantial national capacity for generating clinical evidence, late-stage translation remains a persistent challenge: uptake into routine care can take more than a decade, and research efforts remain concentrated in early translational phases rather than implementation and population-level impact [29]. The CTSA program has emphasized the need for community-engaged, bidirectional, and sustainable strategies to address this T3/T4 gap [30,31]. Yet, researchers often lack an embedded, longitudinal, community-based delivery system capable of supporting real-world implementation. MIH–CP personnel in research roles may help to address this gap.

## Conclusion

MIH-CP clinicians who cross-train as research personnel may offer a compelling and practical solution to several longstanding challenges in clinical research, including limited trial accessibility, inequitable participation, shortages in the research workforce, and the difficulty of implementing evidence-based interventions in real-world settings. By bringing interventions into communities, MIH-CP clinicians decentralize procedures that traditionally require travel and in-person visits. The integration of MIH-CPs into research presents a promising strategy for transforming the generation and implementation of clinical evidence.

## Supporting information

10.1017/cts.2026.10761.sm001 O’Connor et al. supplementary material O’Connor et al. supplementary material

## Supplementary material

The supplementary material for this article can be found at https://doi.org/10.1017/cts.2026.10761.

## Table 1. Long description

A table with 9 rows and 10 columns summarizing the design and outcomes of clinical research studies utilizing CP research staff. The columns are Study, Design, Setting & participants, Type of intervention, MIHCP’s role in intervention, MIHCP’s role in outcome evaluation, Recruitment by MIHCP, Intervention delivery by MIHCP, Outcome evaluation by MIHCP, and Notes. The rows are as follows: Row 1: Pragmatic randomized controlled trial (hybrid design), Adults with COPD recently discharged from a hospital and living in the community, Home visits, Self-management support, Assessment by MIHCP’s routine practice, 12-month hospitalization, 3-day rehabilitation intensity of care, Referral of home-based clinical assessments and interventions, selected participant burden, outcome assessment integrated with routine nursing, Supported participants with limited literacy needed in-home clinical assessment integrated with routine nursing. Row 2: Pre-randomized clinical trial (decentralized), Adults with COPD at high risk for hospitalization, Tele-rehabilitation, In-demand tele-rehabilitation for acute respiratory symptoms, 12-week hospitalization, mortality, 3-day rehabilitation intensity of care, Supported participants with limited literacy needed in-home clinical assessment integrated with routine nursing. Row 3: Observational cohort study, Adults with intellectual and developmental disabilities supported in residential settings, Recruitment, In-demand evaluation for acute differentiated illness, 12-week hospitalization, claims-based mortality, differences in differences analysis, Supported participants with limited literacy needed in-home clinical assessment integrated with routine nursing. Row 4: Observational cohort study, Older adults discharged from hospital rehabilitation after hip fracture, Home visits, Post-discharge transitional care home visits, falls, hospital readmission, discharge, In and 3-day rehabilitation. Row 5: Observational cohort study, Older adults discharged from hospital rehabilitation after hip fracture, Home visits, Post-discharge transitional care home visits, falls, hospital readmission, discharge, In and 3-day rehabilitation. Row 6: Observational cohort study, Older adults discharged from hospital rehabilitation after hip fracture, Home visits, Post-discharge transitional care home visits, falls, hospital readmission, discharge, In and 3-day rehabilitation. Row 7: Observational cohort study, Older adults discharged from hospital rehabilitation after hip fracture, Home visits, Post-discharge transitional care home visits, falls, hospital readmission, discharge, In and 3-day rehabilitation. Row 8: Observational cohort study, Older adults discharged from hospital rehabilitation after hip fracture, Home visits, Post-discharge transitional care home visits, falls, hospital readmission, discharge, In and 3-day rehabilitation. Row 9: Observational cohort study, Older adults discharged from hospital rehabilitation after hip fracture, Home visits, Post-discharge transitional care home visits, falls, hospital readmission, discharge, In and 3-day rehabilitation.

Navigate back to Table 1..

## Figure 2. Long description

A diagram of the research team structure with MIH-CP clinicians. The diagram is divided into three main training categories: Clinical Training, Operational Training, and Research Training. Clinical Training includes EMT/Paramedic Certification, Community Paramedicine Protocols, and Study Specific Clinical Protocols. Operational Training covers EMS Operations, Clinical Oversight, and Clinical Communication. Research Training encompasses Human Subjects Projections, GCP-CITI, Informed Consent, Data Security, and Protocol Fidelity. The diagram also shows the roles of Integrated Community Paramedic Research Personnel, who perform Mobile Interventions such as In-home assessment, Field-based recruitment, On-demand dispatch, Clinical Intervention, and Adverse event monitoring. Additionally, they engage in Traditional Research Roles including Consent, Facility-based recruitment, and Data Capture. The oversight is provided by a Medical Director and a Principal Investigator. A Non-clinical research coordinator is also part of the team, interacting with peers.

Navigate back to Figure 2..
