# Supplementary material for: Research on the road: Partnering with community emergency medical services to expand access to clinical trials
Source: J Clin Transl Sci. 2026 May 29;10(1):e96. doi: 10.1017/cts.2026.10761 (PMC13323617; doi:10.1017/cts.2026.10761)
Supplement: O’Connor et al. supplementary material [file S2059866126107614sup001.docx]

| **Supplemental Table 1: Description of Exemplar Studies** | | | |
| --- | --- | --- | --- |
| **Study Name** | **Rationale and Approach** | **Findings / Study Status** | **Publications (PMID)** |
| **Paramedic Evaluation for Acute COPD Exacerbation (PEACE Trial)** (NCT07072039) | Pragmatic, hybrid decentralized randomized controlled trial evaluating a home-based post-discharge and on-demand acute care intervention for patients recently treated for a COPD exacerbation. The study was designed to address gaps in timely access to care during the high-risk post-discharge period, when patients often experience recurrent symptoms that lead to emergency department visits or rehospitalization. The intervention includes scheduled post-discharge home visits focused on education and self-management, as well as rapid in-home evaluation and treatment for worsening respiratory symptoms. Primary outcomes include acute care utilization, mortality, and patient-reported symptom burden. | Ongoing trial. Early implementation experience demonstrates high operational feasibility, strong protocol fidelity, and successful integration of community paramedics into decentralized research workflows | NA |
| **Healthy at Home for COPD: An Integrated Digital Monitoring, Treatment, and Pulmonary Rehabilitation Trial** (NCT06000696) | This pilot, nonrandomized decentralized trial evaluated an integrated care model combining remote physiologic monitoring, symptom tracking, digital pulmonary rehabilitation, and in-home clinical evaluation for patients with moderate to severe COPD at high risk for exacerbation. The study was designed to test the feasibility and preliminary effectiveness of a hybrid digital and home-based intervention that could support early detection and treatment of symptom worsening while reducing reliance on facility-based acute care. Outcomes included hospitalization, emergency department utilization, mortality, and changes in patient-reported symptom burden over one year. | Across an 18-month enrollment period, 96% of participants completed the study; completion rates for scheduled study instruments ranged from 78–93%. Participants demonstrated mean improvement of 2.48 points in COPD Assessment Test scores from baseline to study completion (p=0.03). Compared to a synthetic control, participants had lower odds of hospitalization (OR 0.67, 95% CI 0.46–0.98) and 30-day readmission (OR 0.38, 95% CI 0.17–0.84). No increases in emergency department utilization, length of stay, or mortality were observed. | **39468649;** **40641609;**  **40676130** |
| **Paramedic Acute Response Approach for Adults with Intellectual and Developmental Disabilities (PARA-AIDD)** | Observational cohort study examining a community-based acute response model for adults with intellectual and developmental disabilities living in supported residential settings. The study was designed to address barriers to timely outpatient evaluation in this population, including communication challenges and limited access to same-day clinical care. The intervention involved rapid in-home assessment and management of undifferentiated acute illness, with outcomes focused on emergency department utilization, inpatient hospitalization rates, and healthcare costs over one year using a difference-in-differences comparison with a usual care cohort. | Ongoing observational analysis | NA |
| **Paramedic Assisted Community Evaluation After Discharge (PACED) Study** | Pilot trial evaluating a transitional care intervention for frail older adults following hospital discharge. The study was designed to assess the feasibility and potential impact of an early post-discharge home visit aimed at identifying clinical instability, medication errors, delirium, and unmet care needs during a high-risk transition period. Primary outcomes focused on implementation feasibility, while exploratory clinical outcomes included 30- and 90-day rehospitalization, emergency department utilization, and detection of post-discharge complications. | In total 92% of enrolled participants received the complete protocol. During post-discharge evaluations, community paramedics identified acute clinical issues in 23% of visits, requested additional clinical or supportive services in 34% of encounters, and detected medication errors in 34% of participants. Compared with a geographically excluded control group, participants had a significantly lower risk of 30-day rehospitalization (RR 0.40, 95% CI 0.19–0.84; p=0.03), with a non-significant trend toward reduced 30-day emergency department visits (RR 0.61, 95% CI 0.37–1.37; p=0.23). | **39030939** |
| **Subacute Rehabilitation at Home (SARAH) Program** | This randomized controlled trial compares post-hospitalization skilled nursing care delivered at home using mobile clinical teams and remote digital monitoring with traditional facility-based skilled nursing facility (SNF) care, evaluating safety, utilization, patient experience, and clinical outcomes. | Ongoing trial. Early implementation experience demonstrates high operational feasibility, and successful integration of community paramedics into decentralized research workflows | **NA** |
